# Local Food System Approaches to Address Food and Nutrition Security among Low-Income Populations: A Systematic Review

**DOI:** 10.1016/j.advnut.2023.100156

**Published:** 2024-03-11

**Authors:** Katharine Garrity, Kathleen Krzyzanowski Guerra, Hannah Hart, Khawlah Al-Muhanna, Emily C Kunkler, Ashlea Braun, Kathryn I Poppe, Kara Johnson, Emma Lazor, Yang Liu, Jennifer A Garner

**Affiliations:** 1 Division of Medical Dietetics, School of Health and Rehabilitation Sciences, College of Medicine, The Ohio State University; 2 John Glenn College of Public Affairs, The Ohio State University; 3 College of Public Health, The Ohio State University; 4 Department of Nutritional Sciences, College of Education and Human Sciences, Oklahoma State University

**Keywords:** fruits and vegetables, nutrition education, nutrition incentive programs, farmers markets, produce prescription programs, community-supported agriculture, mobile markets, farm stands, farm-to-school, food hubs

## Abstract

Food and nutrition insecurity disproportionately impact low-income households in the United States, contributing to higher rates of chronic diseases among this population. Addressing this challenge is complex because of various factors affecting the availability and accessibility of nutritious food. Short value chain (SVC) models, informally known as local food systems, offer a systemic approach that aims to optimize resources and align values throughout and beyond the food supply chain. Although specific SVC interventions, such as farmers markets, have been studied individually, a comprehensive review of SVC models was pursued to evaluate their relative impact on food security, fruit and vegetable intake, diet quality, health-related markers, and barriers and facilitators to participation among low-income households. Our systematic literature search identified 37 articles representing 34 studies from 2000–2020. Quantitative, qualitative, and mixed-method studies revealed that farmers market interventions had been evaluated more extensively than other SVC models (i.e., produce prescription programs, community-supported agriculture, mobile markets, food hubs, farm stands, and farm-to-school). Fruit and vegetable intake was the most measured outcome; other outcomes were less explored or not measured at all. Qualitative insights highlighted common barriers to SVC use, such as lack of program awareness, limited accessibility, and cultural incongruence, whereas facilitators included health-promoting environments, community cohesion, financial incentives, and high-quality produce. Social marketing and dynamic nutrition education appeared to yield positive program outcomes. Financial incentives were used in many studies, warranting further investigation into optimal amounts across varying environmental contexts. SVC models are increasingly germane to national goals across the agriculture, social, and health care sectors. This review advances the understanding of key knowledge gaps related to their implementation and impact; it emphasizes the need for research to analyze SVC potential comprehensively across the rural-urban continuum and among diverse communities through long-term studies of measurable health impact and mixed-method studies investigating implementation best practices.

This trial was registered at PROSPERO as CRD42020206532.

**Statements of significance**

This systematic review addresses a crucial gap by synthesizing and comparing the rigor, outcomes, and implementation of various local food system interventions in the United States aimed at enhancing food and nutrition security. The review uncovers mixed efficacy, identifies research gaps, and offers invaluable insights into outcome measures, implementation barriers, and facilitators. A systematic review of this scale does not exist, and the results can inform local food system interventions targeting individuals living with low incomes.

## Introduction

In 2021, >10% of households in the United States were considered food insecure, meaning that they lacked access to sufficient food for an active, healthy life [1]. Low-income households experience greater rates of food insecurity: 32% of households with incomes below the federal poverty line experienced food insecurity in 2021 [1]. Low-income and food-insecure adults experience higher rates of chronic diseases, such as type 2 diabetes [[2], [3], [4]], heart disease [3,5], mental health conditions, such as depression [6,7], and lower quality of life (QoL) [8,9]. These disparities have been attributed, in part, to poor diet quality. The diets of low-income and food-insecure populations are notably low in fruits and vegetables (FVs) and are of significantly less nutritional quality than the diets of those with higher socioeconomic status [10,11]. It is well-established that insufficient resources—financial and otherwise—are a critical barrier to healthy food access and intake; these resources include lack of transportation [12], high housing and food costs [13,14], being under or unemployed [13], and having low assets [15].

Social safety-net programs, such as the Supplemental Nutrition Assistance Program (SNAP), aim to address food insecurity among low-income individuals. Although food security status is positively impacted by program participation, some analyses have found that poorer diet quality persists, remaining lower among participants relative to their higher-income nonparticipant counterparts [16]. This and other factors have driven greater investment in promoting diet quality among federal food assistance beneficiaries. For example, the Gus Schumacher Nutrition Incentive Program (GusNIP) awards grant funding to nonprofit organizations and government agencies for projects that incentivize FV purchases by SNAP participants [17], and the 2022 White House Conference on Hunger, Nutrition, and Health resulted in explicit calls for “food is medicine” interventions designed to treat or prevent diet-related health conditions via healthy food “prescriptions” [18].

Parallel to the increased focus on nutrition incentives and “food is medicine” interventions, “nutrition security”—a concept that embodies goals related to food security, diet quality, and health equity—has emerged as a necessary national target. The formal definition of nutrition security, as defined by the USDA, is “having consistent access, availability, and affordability of food and beverages that promote well-being and prevent (and if needed, treat) disease, particularly among racial/ethnic minority populations, lower income populations, and rural and remote populations” [19]. Several federal agencies and efforts have committed to moving beyond food security and dedicating resources to combat nutrition insecurity: the National Strategy drafted after the 2022 White House Conference on Hunger, Nutrition, and Health includes multiple pillars focused on nutrition security [18]; the USDA announced a 4-pillar strategic approach to tackle nutrition security [20]; and the 2020–2030 Strategic Plan for NIH Nutrition Research underscores the significance of nutrition security [21].

Research suggests that food-insecure households often sacrifice food quality and variety in favor of quantity (e.g., consuming low-cost, energy-dense, and nutrient-poor foods) [22]. An emphasis on nutrition security warrants improvements in access to nutritious foods and adequate health services to prevent and treat disease, shifting away from a more calorie-centric focus to one that considers the nutritional content of foods [23]. Contrary to colloquial narratives, there is some evidence that food-insecure households prefer more healthful foods (i.e., fruits, vegetables, and high-quality proteins) when given a choice, suggesting that lack of resources, not knowledge or desire for well-being, may be a key factor standing in the way of improved diet quality [24,25]. Given the interconnectedness of diet quality, food insecurity, and chronic disease, the shift to nutrition security holds promise for enhancing innovation in clinical practice and public policy while also advancing health equity.

Addressing food and nutrition insecurity within the United States remains a challenge given the complexity of determinants impacting the food supply, particularly access to nutritious foods. Such intersecting determinants necessitate a systems approach that leverages resources and aligns values across the food supply chain. Short value chain (SVC) models of healthy food access—informally known as local food systems—fit this vision. A food value chain is “a business model in which producers and buyers of agricultural products form strategic alliances with partners along the supply chain to enhance financial returns through product differentiation that advances social or environmental values” and embodies values of “transparency, strategic collaboration, and dedication to authenticity” [26]. Although traditional food supply chains may reflect some of the same operational activities as value chains, these models are unique because of their emphasis on shared missions and operational values. These missions may encompass healthy food access, farm viability, and environmental stewardship [26].

SVC models, such as farmers markets (FMs) and community-supported agriculture (CSA), show promise for influencing key dietary and health outcomes among low-income consumers. FMs, for example, can be a source of healthy food products to SNAP and Special Supplemental Nutrition Program for Women, Infants, and Children (WIC) recipients via the use of the Electronic Benefit Transaction system and incentive program vouchers, respectively. Patronage of FM is associated with increased food security status and increased FV consumption among SNAP participants [27]. CSA participation has resulted in increased vegetable intake [28], decreased frequency of doctor’s visits and expenditures at pharmacies, and improved healthy eating behaviors (e.g., eating salads and preparing dinner at home) [29].

Currently, a systematic review of the literature on all types of SVC models and their various impacts and implementation challenges does not exist. A 2016 systematic review of FM use among low-income consumers found there to be limited use of shared methods and metrics across included studies, limiting the broader understanding of factors that influence FM use [30]. A 2020 scoping review synthesized factors that may impact effectiveness of FV incentive programs for current SNAP participants, although most included studies were conducted at FM [31]. An additional scoping review broadly examined interventions targeting SNAP beneficiaries and their reported impact on diet and nutrition-related outcomes [32]. These reviews, although valuable, occupied a narrower scope, either focusing on a particular low-income group of interest (e.g., SNAP participants) or on 1 type of intervention (e.g., FM-based programs). Given the inherent overlap between and increasingly common integration of SVC models in single interventions, an encompassing review of all SVC models is likely to be helpful in informing future research, practice, and policy.

The purpose of this systematic review was to evaluate whether participation in SVC models of healthy food access influenced food security status, FV intake, total diet quality, and health-related markers and outcomes among low-income households in the United States. The authors also aimed to understand barriers to or facilitators of participant engagement with SVC models in the United States. Given the novelty of the nutrition security construct in the United States, for which no validated measures existed until 2022 [33] and none are yet widely accepted, this review focused on measures of diet quality and food security (among other outcomes) for the quantitative synthesis and interrogated the health equity potential of each model based on qualitative assessments of their accessibility.

## Methods

This review adhered to the PRISMA guidelines. The protocol was developed by 2 coauthors (HH and JAG) in consultation with a library sciences expert and feedback from 1 other author (AB). This protocol was registered with PROSPERO (CRD42020206532) and is available for review.

### Search strategy

Three major topical domains—disparities, SVCs, and food—were used to develop the search. Each domain included a series of keywords and Medical Subject Heading (MeSH) terms. A search strategy was initially prepared for PubMed and adapted for each database by the research librarian. Database-specific indexing terms were included when applicable. Detailed information regarding search terms and search strings is provided in Appendix A. The search was not restricted to the outcomes of interest to afford the most comprehensive search possible. Articles with no relevant outcomes were excluded during the full-text screening process.

Literature published in the English language and in full-text from 2000 to 2020 was searched and accessed via the following electronic databases by 1 author (AB) for upload into Covidence: Agricola, Center for Agriculture and Biosciences International (CABI Abstracts), Cumulative Index of Nursing and Allied Health (CINAHL), Embase, Public Affairs Index, PubMed, Scopus, SocINDEX (a database of sociological scholarship, including social work), and Web of Science. Because of the expansive scope of the search and the extent of relevant literature and reports accessed via these databases, the decision was made not to search for other potential sources of gray literature.

### Selection of articles

Articles were eligible for inclusion if they reported on ≥1 SVC model designed to the following: *1*) minimize the disconnect between farms and consumers by reducing 1 or more “middle” portions of the traditional food supply chain and *2*) leverage local or regional sources of healthy food (e.g., FVs). Such SVC models may include but are not limited to, FM, produce prescription (PRx), mobile market (MM), CSA, farm-to-school (FTS), farm stand (FSt), and food hub (FH) models. Included studies had to either evaluate the effects of these models on ≥1 diet- and health-related outcome (for quantitative studies) or explore barriers to and facilitators of engagement with such models (for qualitative studies). All studies had to focus on households within the United States considered low-income (i.e., ≤185% of the current Federal Poverty Level or as indicated by study authors). We relied on authors’ definitions and descriptions to determine the use of SVC models, and there had to be explicit mention that the produce used in the interventions was sourced locally. If this was unclear, we attempted to contact the authors via email to clarify. If no clarification was received, the article was excluded from the review.

For the quantitative portion of this analysis, randomized controlled trials (RCTs), nonrandomized controlled studies (e.g., controlled prepost studies), and quasi-experimental studies designed to afford causal inference met our inclusion criteria. To enable a comparison between individuals and households, with *some* compared with *no* exposure to the eligible interventions, studies had to include a control or comparison group. Outcomes of interest were food security status (as measured by any version of the USDA Economic Research Service’s food security survey [34], The Hunger Vital Sign (Children’s HealthWatch team, Boston, MA, USA) screening tool [35], or the 1-item screening question included in Safe Environment for Every Kid screener [36]); FV intake (as measured by the National Cancer Institute Fruit and Vegetable Screener [37] or other validated measures); and total diet quality (as measured by the Healthy Eating Index [38] or other validated measures). Secondary outcomes included anthropometric measures (e.g., BMI (in kg/m^2^), weight and waist circumference); biomarkers of health (e.g., blood pressure, total cholesterol, HDL cholesterol, cholesterol ratio, triglycerides, and fasting glucose); health outcomes (e.g., chronic disease diagnoses); and QoL indicators (as measured by WHO QoL-BREF (the abbreviated version of the WHO's 100-item QoL survey) [39], QoL10 [40], or other validated measures).

For the qualitative portion of our analysis, any study that collected data via focus groups or in-depth interviews was included (regardless of the overall study design). Extraction and analysis were focused on the synthesis of insights regarding participant barriers to and facilitators of intervention engagement.

### Screening

Six reviewers (HH, AB, KIP, KG, JAG, and KKG) screened titles and abstracts for eligibility and inclusion using Covidence. Duplicates were removed. Two reviewers were required to screen each title and abstract independently. To settle discrepancies between 2 reviewers, a third reviewer (KG) was consulted to make a blinded, final determination. After the initial screening process, the full text of potentially eligible articles was obtained and screened in more detail. The Population, Intervention, Comparator, and Outcome screening guide is provided in Appendix B.

### Data extraction

For included studies, relevant outcomes from quantitative studies or findings from qualitative studies were compiled into an Excel document. Data from all included studies were extracted by 9 team members (KG, KKG, KJ, KIP, ECK, EL, YL, KA-M, and HH) and checked for accuracy and completeness by 2 team members (KG and KKG). Extracted data included the name of the first author, year of publication, methodology (qualitative, quantitative, or mixed method), study design, study objective, type of SVC intervention, intervention components, geographic area (rural compared with urban), sample size, author criteria for (or definition of) low-income, and key quantitative and/or qualitative findings.

### Risk of bias assessment

The risk of bias was assessed for each study. Quantitative studies were appraised using the National Institute of Heart, Lung, and Blood Quality Assessment Tools for Controlled Intervention Studies, Observational Cohort Studies, and Case-Control Studies [41]. Quality rating of included studies was completed independently by 1 of 7 reviewers (KG, KKG, KA-M, KIP, KJ, ECK, EL, and YL). Secondary reviews—i.e., detailed checking and confirmation of each criterion and overall score—were completed by 3 reviewers (KG, KKG, and KA) such that all studies were reviewed by ≥2 team members. Disagreements were resolved via discussion between the primary and secondary reviewers, with a third author serving as the tiebreaker when necessary. Qualitative studies were appraised using The Standards for Reporting Qualitative Research (SRQR) devised by O’Brien et al., (2014) [42].

### Data synthesis

Extracted data were reviewed iteratively by the author team to produce a comprehensive summary of included studies and their attributes in table form. Upon completion of the table (Table 1), data synthesis involved additional iterative, team-based reviews of the extracted content over a series of several intensive meetings to come to consensus on key insights across the following 6 domains: *1*) the types of data and study designs used to evaluate SVCs to date, *2*) the methodologic quality of studies to date, *3*) intervention subtypes and key intervention characteristics, *4*) the role of nutrition education (given the field’s historic focus on education as a key strategy for individual behavior change), *5*) whether the studied interventions demonstrated impact across the outcomes of focus, and *6*) what we know to date about barriers to and facilitators of program engagement with the studied SVC models. For the reader’s convenience, the results section was organized accordingly, and all insights were summarized narratively. For articles reporting qualitative data, the manuscripts were uploaded into NVivo (Lumivero) for coding of themes regarding barriers to and facilitators of SVC engagement by target participants.

**TABLE 1:** Characteristics of the short value chain interventions included in the systematic review

Farmers market (FM) (*n* = 18) – food markets at which local farmers sell directly to customers												
Quantitative studies							Outcomes of interest					Quality grade^3^
Author, date	Study design	Study objective	Intervention	Duration	Sample	Setting	FS	FV	DQ	AM	BoH	
Anderson et al., 2001 [43]	Quasi-experimental nonequivalent control group with 4-arms	Determine the effect of Michigan FMNP in 1 county on FV consumption behavior	Subjects assigned to 1 of 4 groups: 1. FV education 2. FM coupons 3. Both 4. No intervention	Established FMs; pretest and posttest questionnaires administered 2 mo apart	*n* = 564 WIC participants at the pretest; *n* = 465 at the posttest (100% female, 43% Black, 49% White, 7% Other racial groups, mean age of 30 y)	Flint, MI (urban)		+; for education and coupon arms				Poor
1Di Noia et al., 2017 [53]	4-arm RCT	Evaluate the effects of the WIC Fresh Start education program	Participants were stratified based on FMNP voucher receipt and randomly assigned to either WFS online lesson or existing WIC online health education	Established FMs; pretest then posttest 2 wk after the lesson. Follow-up assessments conducted at 3 and 6 mo posttest	*n* = 744 WIC participants randomly assigned (100% female, 59% Hispanic, 30% Black, 9% White or Other, 2% 2 or more races, with a mean age of 29 y)	NJ (urban)		NE				Fair
Herman et al., 2008 [46]	Quasi-experimental nonequivalent control group with 3-arms	Evaluate whether an economic subsidy for FV would increase FV consumption for postpartum WIC participants	Bi-monthly produce vouchers, redeemable at FM (group 1) or supermarkets (group 2) over the 2-mo period; the control group received minimal nonfood incentive (group 3)	6-mo intervention with data collected at 2 mo preintervention (group 1 and 2 only), baseline, 2 mo after baseline, end of 6 mo intervention, and 6 mo following the intervention	*n* = 602 WIC participants enrolled; *n* = 451 WIC participants completed the study (100% female; 89% Hispanic, 6% Black, 3% non-Hispanic White, 2% Asian American, with a mean age of 28 y)	Los Angeles, CA (urban)		+; for both intervention groups, sustained 6 mo postintervention				Poor
Johnson et al., 2004 [51]	Quasi-experimental pretest-posttest with control group	Determine if Seattle Farmers’ Market Nutrition Pilot Program increased FV intake among older adults (age 60+) who received FM baskets	Biweekly delivery of FV baskets	5-mo program	*n* = 152 older adults at baseline; *n* = 131 older adults at follow-up (73% female, 69% White, 23% Black, 2% Hispanic, 2% Asian-Pacific Islander, 1% American Indian-Alaskan Native, and 4% Unknown)	Seattle, WA (urban)		+				Poor
Stallings et al., 2016 [54]	Quasi-experimental time series with control group	Evaluate the impact of FMNP among WIC participants, including mothers and children	FMNP participants received $30 in coupons for fresh produce at eligible FM; the Non-FMNP group received WIC standard-of-care	3-mo study with 3 survey time points: baseline, 1-wk, and 4 wk postenrollment	*n* = 149 WIC participants (99% Black, 77% SNAP or TANF recipients, 38% were between ages 25–31 y); response rates were 88.6% at 1-wk and 81.9% at 4-wk	Atlanta, GA (urban)		NE				Fair
Weinstein et al., 2014 [50]	2-arm RCT	Test the impact of distributing FM coupons and provision of education on FV purchase and consumption in overweight patients with T2DM	Participants were randomly assigned to standard of care or $6 in FM coupons and a 1-h education session on the benefits of FV intake	Established FMs (Green Markets); survey data collected at baseline and 12 wk	*n* = 79 enrolled participants with T2DM; *n* = 78 analyzed participants with T2DM (69% female, 31% male; 49% Hispanic, 33% Black, 3% White, 15% Other or >1 race, with a mean age of 56 y)	The Bronx (NYC), NY (urban)		NE; Non-significant increase in FV intake in the intervention group		NE; Non-significant decrease in BMI across both groups	NE; Non-significant decrease in HbA1c across both groups	Fair
Qualitative studies							Barriers			Facilitators		
Cohen et al., 2019 [60]	Focus groups	To examine participants' motivations for using DUFB, facilitators/barriers to DUFB use, and intervention acceptability	Waiting room-based informational intervention encouraging DUFB use at a local FM	Established FM; 1-time focus groups postintervention	*n* = 5 focus groups, 26 SNAP-enrolled participants; 77% female, 65% Black/non-Hispanic, 27% White/non-Hispanic, 50% disabled, with a median age of 45 y	MI (urban)	Lack of transportation; limited market locations/hours; seasonal limitations of FM produce; precludes the efficiency of 1-stop shopping for groceries; persistent confusion regarding incentive use among a small subset of the sample			Desire to eat more healthfully; stretching SNAP benefits; higher quality produce; unique market environment, relationships with farmers		Fair
Colasanti et al., 2010 [79]	Focus groups *(mixed methods – only qualitative data included)*	Assess potential differences in perceptions of FMs and shopping behavior between demographic groups	Use (or nonuse) of FMs	Established FMs; 1-time focus groups	*n* = 7 focus groups, a total of 63 participants; a mix of racial and ethnic backgrounds (e.g., Caucasian, non-Hispanic, Asian, Hispanic, African-American, Latina, Middle Eastern and Arab American)	MI (3 urban and 4 rural sites)	Poor awareness of FM locations, hours, season of operation, and accepted methods of payment; poor marketing; time constraints; inconvenient hours/ locations; inconsistent acceptance of SNAP across locations; perception of FMs as unwelcoming to families of color; language barriers			Walkability, visibility, ability to support local farmers		Fair
Headrick et al., 2020 [75]	Semistructured interviews *(mixed methods – only qualitative data included)*	Identify the facilitators and barriers of the Maryland Market Money Program (MMM) and generate recommendations for the implementation of FM incentive programs elsewhere	Customers spending their SNAP, WIC FV Checks, or FMNP vouchers were eligible to receive $5 per participating market per day	Established FMs distributing MMM; semistructured interviews spanning 2 FM seasons	*n* = 48 interviews and group interviews (2–4/group, 58 total nutrition assistance beneficiaries); 83% of customers surveyed and interviewed were female with a mean age of 51.5 y	MD (urban); 2 FMs in Baltimore City, 1 FM in Prince George's County, 1 FM in Montgomery County	Transportation; poor mobility (for seniors); confusion regarding eligible purchases under MMM; inability to turn in >1 FMNP receipt per week; poor marketing; MMM funds running out			Use of 1-dollar, universal tokens across markets; increased spending power; positive change in shopping behaviors; reduced stigma; ability to support farmers		Good
Cotter et al., 2017 [64]	Focus groups	Examine how low-income, minority communities in Washington, DC, perceive local FM and CSA programs	Use (or nonuse) of FMs	Established FM with planned CSA and nutrition education component; 1-time focus groups	*n* = 4 focus groups, 28 total participants; 86% female, 86% Black, 4% Hispanic, 4% Asian, 4% American Indian, and 4% Other race, 89% received Medicaid, 64% SNAP participants, 11% WIC participants, the modal income of <$15,000, the mean age of 62.5 y	Washington DC (urban)	Cost of produce at FM; transportation			Quality of FM products is superior to grocery stores and food pantries		Good
1Di Noia et al., 2017 [66]	Focus groups	Explore perceived barriers and facilitators to purchasing FV at FMs and reactions to a planned WIC nutrition education lesson	Participants received an overview of a planned nutrition education lesson to promote FM use among women enrolled in WIC	1-time focus groups	*n* = 13 focus groups, a total of 54 WIC participants; 45% non-Hispanic Black, 44% Hispanic, 70% English-speaking, 65% unemployed, 86% with a high-school education or less, and median age of 27 y	Inner-city area of NJ (urban)	Transportation issues; not knowing the location of the markets; inconvenient market hours and locations; time constraints; limitations of FMNP, such as funding constraints; not being in the habit of eating healthfully					Good
Garner et al., 2020 [55]	Focus groups	Explore factors affecting access to and use of DUFB, an FM program that doubles SNAP benefits for use toward the purchase of local FV	SNAP participants receive a dollar for every dollar spent on in-state grown FV at participating markets ($10 match limit per market visit in Utah, $20 match limit per visit in New York)	Established seasonal program; 1-time focus groups	*n* = 9 focus groups (*n* = 4 in NY, all program users; *n* = 5 in UT, a mix of program and nonprogram users), a total of 62 SNAP participants; NY participants were 79% female, 95% White, 95% non-Hispanic, with a mean age of 46 y; UT participants were 90% female	2 counties in upstate NY (1 urban, 1 rural); 5 counties in UT (4 urban, 1 rural)	Poor marketing and insufficient directions for program use; issues of inconvenience, such as lack of available free parking near participating markets, poor transportation options, and location and timing of markets			Ease of program use; market access/location (in some cases); placement of FV vendors at the front of the market; vendor availability		Good
Grace et al., 2007 [74]	Interviewer-led surveys with open-ended interview questions *(mixed methods – only qualitative data included)*	Investigate SNAP participants’ perceptions of FMs in Portland, Oregon	Use of SNAP benefits at FMs	Established FMs; 1-time interviews	*n* = 108 SNAP participants; 74% female, 65% represented family households, between ages of 18–65 y	Portland, OR (urban)	Unaware of ability to use EBT card at FMs; perception of higher prices than at food stores; limited hours and locations; lack of variety and availability of foods year-round; poor usability; lack of value or product deals			Location; subsidy via WIC FMNP; prices (general products, not specifically FV); social benefits		Fair
Larimore, 2018 [58]	Semistructured interviews	Examine the process through which cultural barriers are created and persist in 2 urban SNAP-accepting FMs located in or in very close proximity to food deserts	Use of SNAP benefits at 2 urban FMs (Southside and North market)	Established FMs; field observations, informal interviews, and formal, semistructured interviews collected over 5 mo	*n* = 12 participants; Southside market customers were primarily White and middle class, whereas North market customers were largely non-White and working to lower-middle class	Southeastern United States (urban)	Lack of transportation (i.e., no bus line); social isolation and stigma; perceived lack of wider community recognition (insufficient support/marketing by city); perceived lack of healthy eating education; lack of knowledge related to location and/or acceptance of EBT			Tradition/ familiarity with the market; desire to support community; produce delivery available, if immobile; FV affordability; perceived storage life of FV; experience with home gardens; perceived health benefits of local FV		Good
Masci et al., 2020 [59]	Focus groups and in-depth interviews	Evaluate the implementation of an FM−based FV incentive program; describe the use of the program, how DUFB affects purchasing, and program barriers and facilitators	SNAP participants receive a dollar for every dollar spent on in-state grown FV at participating FMs and MMs (≤$20/visit at FMs or $10/d at MM)	Established FMs and MM; 1-time focus groups and interviews	*n* = 4 focus groups and *n* = 6 phone interviews, a total of 36 SNAP participants; 75% female, 67% White, 70% unmarried, 42% held a college degree, 69% reported income of <$20,000, and 53% reported low or very low food security and age range of 25-86 y	Western NY (4 rural and 5 urban FMs; 1 MM)	Unaware which FMs accept DUFB; limited SNAP benefits preclude full desired use of DUFB; inability to transport or store FV; SNAP-related stigma; barriers with token system (locations running out of tokens, equipment malfunctioning); FMs not carrying desired items; vendors not participating in DUFB; low quality of FV; vendors not understanding program implementation			Current token system was easy to use; helpful staff, convenient market location, helpful vendors, access to transportation to and from the market, and even pricing (i.e., pricing rounded to the dollar to limit the need for change); the desire to support local farmers		Good
McGuirt et al., 2014 [61]	In-depth interviews	Learn factors related to locally sourced food procurement among women of reproductive age	Use (or nonuse) of local food sources, primarily FMs and FSts	Established markets and stands; 1-time in-depth interviews	Western North Carolina (*n* = 23): 59% Black, 51% unemployed, 51% SNAP participants, 14% WIC participants, 59% between ages of 20–29 y; Eastern North Carolina (*n* = 37): 100% White, 57% unemployed, 100% WIC participants, 52% between ages 20–29 y	NC (rural)	Inconvenient locations; grows their own garden; markets/stands do not accept SNAP/EBT; unaware of market/stand locations; perception of higher cost; not part of food shopping routine; time constraints; distrust of produce sold; poor familiarity with FM experience			Preference to buy local and fresh produce; convenient location; increased opportunities for socialization; organic options; perception of lower cost; taste of produce; ability to buy in bulk		Fair
Savoie Roskos et al., 2017 [70]	Semistructured interviews	Identify benefits and barriers to using an FM incentive program among program participants	Participants received regular (no SNAP spending required) or matched incentives ($1 in FM currency for every $1 in SNAP benefits spent at FM)	8-wk incentive program at established FM; 1-time semistructured interviews	*n* = 14 SNAP participants; 100% White, 71% female, with a mean age of 37 y	Northern UT (urban)	Participants were unaware that SNAP/EBT was accepted at FM; inconvenient market hours and days of operation			Increased FV exposure for children; improved FV intake; the opportunity to build local connections and purchase locally grown foods; incentives helped offset the cost of FV		Good
Wetherill and Gray, 2015 [62]	Focus groups	Examine barriers to FM use by SNAP consumers receiving TANF	Use (or nonuse) of SNAP benefits by TANF recipients at Oklahoma's largest FM	Established FM; 1-time focus groups	*n* = 8 focus groups, 64 total SNAP participants receiving TANF; 98% female, 69% single heads of households, 55% reported ≥2 dependent children living in the home, with a mean age of 27 y	Tulsa, OK (urban)	Unfamiliarity with FM products and locations, including SNAP/EBT acceptance; perception of less FV variety; perception that FV is more expensive; limited hours of operations; perceived lack of belonging or fitting typical FM shopper demographic; questionable quality of FM produce; distrust because of perceived lack of regulation; incompatible with typical eating habits; greater complexity of the FM's centralized EBT system			FM offer higher quality and fresh produce		Good

Produce prescription program (PRx) (*n* = 7) – a clinic-community collaboration in which a healthcare representative refers patients to receive free or discounted fruits and vegetables												
Quantitative studies							Outcomes of interest					Quality grade^3^
Author, date	Study design	Study objective	Intervention	Duration	Sample	Setting	FS	FV	DQ	AM	BoH	
2Stotz et al., 2019a [73]	Quasi-experimental pretest-posttest with control group (mixed methods – quantitative and qualitative data included)	Measure the effect a 12-wk supplemental produce and eLearning nutrition education program has on diet quality, food security status, and select clinical outcomes of safety-net clinic patients	Multimodal; the intervention group received a 12-wk program inclusive of 10 eLearning nutrition education lessons and a weekly bag of produce; the control group received neither	12-wk intervention; data collected pre and postintervention	*n* = 26 SNAP-Ed-eligible participants; 69% female, 60% White, the mean age of 47 y, high prevalence of diabetes (46.2%), hypertension (81.0%), and obesity (88.5%) (*n* = 26). 50% of the intervention group and 55% of the control group reported food insecurity during the previous 30 d	South GA (rural)	NE		NE	NE; no significant differences in BMI between groups	NE; no significant differences in lipid panel, fasting blood glucose, HbA1c, and BP between groups	Poor (Note: poor quality grade reflects the quantitative portion of the study only)
Qualitative studies							Barriers			Facilitators		
Cahill et al., 2020 [67]	Telephone interviews	Qualitatively assess constraints on program participation, barriers to maintaining a healthy diet among participants, and participant capacity to sustain behavior change during and after the program	PRx within a primary clinic; 4 wks of FV prescriptions, monthly nutrition education, and cooking skill classes	6-mo intervention (July–December); 1-time postintervention interviews	*n* = 32 participants; 72% female, 91% Black or Caribbean American, 100% had BMI >30 and ≥1 associated chronic condition	Atlanta, GA (urban)	Continued financial constraint despite vouchers off-setting FV cost; lack of time to prepare produce; lack of transportation					Fair
Esquivel et al., 2020 [77]	Telephone interviews *(mixed methods – only qualitative data included)*	Evaluate the feasibility of a community-based pediatric PRx, including facilitators of and barriers to participation	3-mo program; eligible children’s parents were provided with vouchers valid for $24/mo to use on fresh FV at weekly FM	Distribution of vouchers took place between July 2018 and April 2019; 1-time postintervention interviews	*n* = 33 participants; children aged 2–17 (mean age of 8) y who had "poor nutrition" based on growth assessment or BMI percentile	Waianae, HI (rural)	Participants only purchased FV they were familiar with or had recipes for			Increased affordability and accessibility to FV; ease of program use; increased communication with clinicians; positive child and family lifestyle changes; enjoyment attending FM; ability to buy and eat local FV		Poor
Forbes et al., 2019 [78]	Telephone interviews *(mixed methods – only qualitative data included)*	Preliminary evaluation of a student-designed, modified PRx that integrated a community-based, month-long educational curriculum	Multimodal; 6-wk program included weekly PRx for FV at FMs and weekly nutrition education modules	PRx redeemable at established FMs; follow-up interviews 3 y postintervention	*n* = 9 families; 4 with men and 5 with women as head of household, 6 identified as Black, 6 had <$40,000 total income, 2 had income between $50–60k; all were primary care patients “at risk” of chronic illness or metabolic disease with poor reported access to FV	Hershey, PA (urban); Harrisburg, PA (urban)	Limited ability to continue eating healthfully after the program ends because of poor affordability			Ease of program use; enjoyable and educational interactions with medical student mentors		Poor
1Schlosser et al., 2019a ("You Guys Really Care About Me…") [71]	Semistructured interviews	Understand participants' experience using a PRx for adults with hypertension seen at 3 safety-net clinics	Monthly, providers checked BP, provided tailored nutrition counseling for BP control, and prescribed free fresh FV vouchers ($40/mo x 3 mo), redeemable at participating FMs	3-mo PRx available across 3 clinics and 20 FMs; 1-time postintervention semistructured interviews conducted 3–8 mo postintervention	*n* = 23 hypertensive participants; 78% female, 100% Black, 43% SNAP participants, 36% with high-school education or below, with a mean age of 62 y	Cuyahoga County, OH (urban)	Economic insecurity shaped program participation and limited ability to maintain behavior change; lack of reliable transportation and money for gas			Education and care provided by clinicians; group education created positive social space; knowledge gained during education sessions regarding food preparation and storage methods; financial support improved access to and intake of FV; ability to share produce and program experiences with family members		Good
1Schlosser et al., 2019b (“The coupons and stuff just made it possible”) [68]	Semistructured interviews	Understand how economic constraints influence participants’ experience using a PRx for adults with hypertension	See Schlosser et al., 2019a	See Schlosser et al., 2019a	See Schlosser et al., 2019a	Cuyahoga County, OH (urban)	Limited access to reliable and affordable transportation; limited and unstable incomes leading to significant economic insecurity that made it difficult to participate in the PRx; lack of basic food preparation tools			Increased access to and affordability of FV; individual motivation for healthy eating		Good
2Stotz et al., 2019b [73]	Focus groups (mixed methods – quantitative and qualitative data included)	Explore the experiences of safety-net clinic patients who engaged in a 12-wk supplemental produce and eLearning nutrition education program	Multimodal; 12-wk program included 10 eLearning nutrition education lessons and a bag of produce weekly	1-season intervention; focus groups preintervention and postintervention with intervention group only	See Stotz et al., 2019a	South GA (rural)	Lack of produce variety; time constraints; lack of knowledge and skills required for produce preparation; challenges with transportation to pick up weekly produce; technical issues with smartphone-based eLearning modules			Sense of community and togetherness; program benefits reaching beyond participant to family and friends; program participation leading to grocery bill reduction and health improvements		Fair (Note: Fair quality grade reflects qual portion of study only)

Mobile market (MM) (*n* = 5) – produce is aggregated, typically in a single large vehicle, and transported directly to various neighborhoods for short-term sale												
Quantitative studies							Outcomes of interest					Quality grade^3^
Author, date	Study design	Study objective	Intervention	Duration	Sample	Setting	FS	FV	DQ	AM	BoH	
Leone et al., 2018 [45]	Cluster RCT	Evaluate the impact of a mobile produce market, Veggie Van (VV), on FV consumption in lower income communities in North Carolina	VV MM was held weekly during the 6-mo intervention period, with half-priced produce, complemented by nutrition education	Established MM (VV) program; RCT reports on the effect of 6-mo of exposure to VV	*n* = 201 enrolled, *n* = 142 completed follow-up; 96% female (*n* = 142), 65% Black (*n* = 139), 67% not married (*n* = 141), 62% received some form of government assistance (*n* = 124), 54% had an annual income of <$30,000 (*n* = 124), mean age was 46 y (*n* = 140)	4 counties in NC (rural or urban classification unclear)		+				Fair
Gans et al., 2018 [52]	Cluster RCT with 8 intervention and 7 control sites	Evaluate the efficacy of the “Live Well, Viva Bien” program	Multimodal intervention that included discounts, mobile fresh FV markets with nutrition education	1-y long intervention; included baseline, 6-mo, and 12-mo surveys	*n* = 1597 participants completed the baseline survey (73% female, 48% White, 17% Black, 20% Mixed race, 15% Other race, 54% Hispanic, 82% food assistance recipients); the intervention group had 83% follow-up at 6 mo, 78% at 12 mo; the control group had 87% follow-up at 6 mo, 82% follow-up at 12 mo	Providence, RI (urban)		+				Good
**Qualitative studies**							Barriers			Facilitators		
DeWit et al., 2020 [56]	Focus groups	Examine barriers to FV consumption among food-insecure families	Primary care providers at a large clinic dispensed a $5 FV voucher for community MM (with schedule and educational brochures) to food-insecure families during a clinic visit	PRx pilot program leveraging a MM operational from April-October 2017; 1-time focus groups postintervention	*n* = 6 focus groups, total of 29 participants; 90% female, 41% Hispanic/Latino American, 38% Black, 17% White, 4% Multiracial, 38% aged 30–39 y	Midwestern city in the United States (urban)	Lack of transportation; insignificant voucher dollar amount; highly variable MM schedule; unavailability of nonproduce items requiring an extra shopping trip on top of MM stop					Good
Horning et al., 2020 [63]	Focus groups	Inductively understand the impact of the full-service Twin Cities MM, a mobile grocery store that visits underserved, low-income communities	MM	Established MM; 1-time focus groups	*n* = 4 focus groups, a total of 29 participants; 85% female, 38% identified as a diverse person of color, 83% receiving ≥1 form of economic assistance, 81% aged 51 y or older	Minneapolis-Saint Paul, MN (urban)				Door to door service decreased transportation and mobility barriers; weekly schedule minimized need to stock up/waste FV; improved affordability of FV, especially thanks to Market Bucks program (matched SNAP ≤$10 for FV); perceived safety relative to food stores; perceived health improvements		Good
Haynes-Maslow et al., 2015 [65]	Focus groups	Examine the relative strengths and weaknesses of MMs, EBT at FMs, and community gardens as perceived by low-income individuals	Use (or nonuse) of MMs, EBT at FMs, and community gardens	Established, seasonal programs; 1-time focus groups	*n* = 13 focus groups, 105 a total participants; 71% Black, 74% female, 71% had an annual household income of <$20,000, 61% used SNAP or other government assistance, and 53% had a high-school education or less	NC (urban); counties: Buncombe, Durham, Guilford, Orange, and New Hanover	Affordability (even with SNAP benefits); lack of cooking and nutrition knowledge; personal food preferences; perishability; community safety concerns; poor accessibility in terms of hours/days of operation and locations; and perceived stigma of using EBT at FM					Good

Community-supported agriculture (CSA) (*n* = 4) – community members buy a share of a farmer’s produce and receive portions of the harvest regularly throughout the growing season												
Quantitative studies							Outcomes of interest					Quality grade
Author, date	Study design	Study objective	Intervention	Duration	Sample	Setting	FS	FV	DQ	AM	BoH	
Berkowitz et al., 2019 [49]	2-arm RCT	Determine whether a subsidized CSA share would improve diet quality in individuals at high risk of diet-related illnesses	Established CSA; intervention group was given $300 toward a "full" ($690) or "small" ($480) share per growing season vs. $300 cash to the control group	2 growing seasons (24 wk each) spanning May 2017 to December 2018	*n* = 122 participants at high risk of diet-related illnesses (81% female, 90% White, 3% Black, 2% Hispanic, 6% Asian/multi-/Other race, 39% SNAP participants, 37% food insecure, median income was 146% of the federal poverty guideline, with a mean age of 50 y); 14 in the intervention group, 8 in the control group lost to follow-up	Franklin County, MA (rural)	NE; insignificant decrease in food insecurity between the intervention group	+; total veg and total fruit HEI sub-scores are significantly higher between the intervention group	+; significantly higher total HEI score between the intervention group	NE; insignificant decrease in weight between the intervention group	+; a significant decrease in diastolic BP between the intervention group	Fair
Quandt et al., 2013 [48]	2-arm randomly assigned, controlled feasibility study	Test if providing a summer's CSA share and supportive programs would be associated with increased household FV variety and consumption in low-income, minority families	Multimodal; intervention participants were provided with a CSA share once/wk for 16 wk, provided 2+ recipes, and offered 5 evening education and skill-building sessions	Weekly CSA share for 16 wk from May to August 2012; data collected at baseline and postintervention	*n* = 50 enrolled, n = 44 reached at follow-up; 100% female, 96% Black, 4% White or other, 94% unmarried, 82% had 12–14 y education, with a mean age of 37 y(*n* = 50)	Forsyth County, NC (urban)			NE			Fair
Qualitative studies							Barriers			Facilitators		
1McGuirt et al., 2019 [72]	Focus groups (mixed methods – only qualitative data included)	Examine the challenges and opportunities related to CSA pickup location	15- to 24-wk summer CSA share (price subsidized by 50%) combined with tailored nutrition education	Established CSAs with subsidy provided for intervention trial; 1-time focus groups	Not reported	NY, NC, VT, WA (rural or urban designation unclear)	Inconvenient pickup locations; parking difficulties at pickup sites; time constraints making pickup more difficult			Convenient pickup locations such as schools, homes, and central areas; flexible pickup times; building relationships with farmers		Fair
1White et al., 2018 [69]	Focus groups	Examine perspectives on food access among low-income families that participated in a subsidized CSA	15- to 24-wk summer CSA share (price subsidized by 50%) combined with tailored nutrition education	Established CSAs with subsidy provided for intervention trial; 1-time focus groups at the end of the first CO-CSA season	*n* = 14 focus groups with a total of 53 participants; 100% had 1+ child(ren), 94% female, 64% White non-Hispanic, 19% Black, 17% Other or Unknown race, 45% employed, 67% with annual household income <$35,000	NY, NC, VT, WA (rural or urban designation unclear)	Challenges related to pick up sites (e.g., distance, time constraints, parking, poor organization); inability to self-select FV in CSA boxes; poor FV quality (e.g., occasional presence of bugs, slugs, and mold); FV spoilage; difficulty using unfamiliar produce; lack of flexible payment methods			Convenient pickup locations with easy parking and efficient site organization; friends/family assisting with pickup; flexibility of pickup site and time; subsidized price of CSA; ability to choose CSA share sizes		Good

Farm-to-school (FTS) (*n* = 3) – sourcing of locally grown produce for use in school-based meals and snacks												
Quantitative studies							Outcomes of interest					Quality grade
Author, date	Study design	Study objective	Intervention	Duration	Sample	Setting	FS	FV	DQ	AM	BoH	
2Gibson et al., 2014 [76]	Quasi-experimental pilot (mixed methods – quantitative and qualitative data included)	Evaluate the overall nutrient content and differences in quality of FTS lunches compared with traditional lunches provided by a Head Start school	Multimodal; FTS program (traditional lunches Monday- Thursday, FTS lunches on Friday), weekly nutrition education lesson, once-monthly farm tables offering parents local FV and recipes to take home	22-wk implementation of FTS program at year-round Head Start preschool; direct observations of dietary intake performed on randomly selected 20–25% sample of students weekly	*n* = 85 ethnically diverse, low-income students between 3–5 y of age	Midwestern city in the United States (urban)			+; for fiber, % calories from protein, sugar, and % calories from fat			Poor (Note: Poor quality grade reflects the quantitative portion of the study only)
Kropp et al., 2018 [44]	Quasi-experimental pretest-posttest with control group	Examine the effects of serving locally procured produce as part of an FTS program on the selection and consumption of FV among NSLP participants	FTS program	Established FTS program; 6-mo intervention period, with plate waste data collected at baseline and endpoint	6 elementary schools (3 treatment and 3 control); participating children were 47% White, 31% Black, 10% Hispanic, 5% Asian, and 8% Other race(s)	Alachua County, FL (urban)		+				Fair
Qualitative studies							Barriers			Facilitators		
2Gibson et al., 2014 [76]	Focus groups and semistructured interviews (mixed methods – quantitative and qualitative data included)	Assess the perceptions of the FTS program among parents, teachers, administrators, and food service staff, along with the challenges to/ barriers to adopting and sustaining the program	See Gibson et al., 2014a	22-wk implementation of FTS program at year-round Head Start preschool; 1-time focus groups and interviews	*n* = 3 focus groups with 17 parents, 88% female, average age of 32 y; *n* = 2 focus groups with a total of 10 teachers, 100% female, between the ages of 20 and 60 y, average of 7 y of teaching; *n* = 4 interviews with administrators and food service staff, no demographics	Midwestern city in the United States (urban)	Difficulty getting children to try new and unfamiliar foods; lunch items did not reflect the ethnic diversity of enrolled children; cost of FV and lack of accessible FMs made it challenging for parents to implement changes (i.e., increased FV intake) at home			Farm table presentations helped improve parent awareness of the program and encouraged them to try program recipes at home; hands-on cooking classes for the children encouraged them to help with cooking at home		Fair (Note: Fair quality grade reflects the qualitative portion of the study only)

Farm stand (FSt) (*n* = 1) – a small market at which goods from a single farm operation are sold in a manned or un-manned manner									
Author, date	Study design	Study objective	Intervention	Duration	Sample	Setting	Barriers	Facilitators	Quality grade
Hu et al., 2013 [57]	In-depth interviews, focus groups, and participant observation	Identify strategies to promote locally grown produce from an urban food security project, Produce From the Park (PFP), an urban farm	Urban farm/FSt	Established urban farm; 1-time focus groups and interviews with community residents; 1–2 interviews with community organization representatives	*n* = 8 community organization representatives, 38% Black, 63% between 30–49 of age, and 38% >50 y of age; *n* = 2 focus groups, 16 total participants, no demographics; *n* = 7 interview participants, 86% Black, 14% 30–49 y of age, and 71% >50 y of age	Mid-Atlantic City in the United States (urban)	Time constraints (no time for FV preparation); lack of knowledge regarding healthy foods; lack of interest in trying healthy foods and changing current behaviors; competing priorities leading to the desire for fast, easy, and cheap food preparation; low awareness of PFP program		Fair

Food hub (FH) (*n* = 1) – a centralized retail operation at which goods from multiple farm operations are aggregated for marketing and sale to community members												
Quantitative studies							Outcomes of interest					Quality grade
Author, date	Study design	Study objective	Intervention	Duration	Sample	Setting	FS	FV	DQ	AM	BoH	
Sharpe et al., 2020 [47]	Quasi-experimental evaluation with matched comparison community	Evaluate an FH impact in a low-income, low-access setting on dietary intake, behaviors, and perceptions	FH access	Evaluation at baseline, posttests at 6 and 18 mo after FH’s opening	*n* = 527 (79% female, 92% Black, 5% White, 3% >1 race and all others, 31% <high-school diploma, and 30% with very low food security); 17% attrition at 6 mo, 25% at 18 mo	Southeastern United States (urban)		-; Controls increased FV intake relative to FH shoppers	NE	NE (for BMI)		Good

Abbreviations: AM, anthropometric measures; BMI, body mass index; BoH, biomarkers of health; BP, blood pressure; DQ, total diet quality; DUFB, Double Up Food Bucks; EBT, electronic benefits transfer; FMNP, farmers market nutrition program; FS, food security status; FV, fruit and vegetable; HbA1c, hemoglobin A1c; HEI, Healthy Eating Index; NSLP, national school lunch program

RCT, randomized controlled trial; SNAP, Supplemental Nutrition Assistance Program; T2DM, type 2 diabetes; TANF, temporary assistance for needy family; WIC, special supplemental nutrition program for women, infants, and Children; CO-CSA, cost-offset community supported agriculture.

+ = indicates a significant positive effect, NE = indicates no significant positive or negative effect, - = indicates a significant negative effect.

1 Indicates that there are 2 distinct articles depicting 1 study (Di Noia et al., 2017 and Di Noia et al., 2017; Schlosser et al., 2019 and Schlosser et al., 2019; McGuirt et al., 2019, and White et al., 2018).

2 Indicates that this is a mixed-methods study in which both the quantitative and qualitative data met inclusion criteria and, therefore, are included twice throughout the table despite data being extracted from just 1 article (Stotz et al., 2019 and Gibson et al., 2014).

3 Risk of bias assessments were completed for all articles. Quantitative studies were appraised using the National Institute of Heart, Lung, and Blood Quality Assessment Tools for Controlled Intervention Studies, Observational Cohort Studies, and Case-Control Studies. Qualitative studies were appraised using The Standards for Reporting Qualitative Research devised by O’Brien et al., (2014).

## Results

**FIGURE 1:**
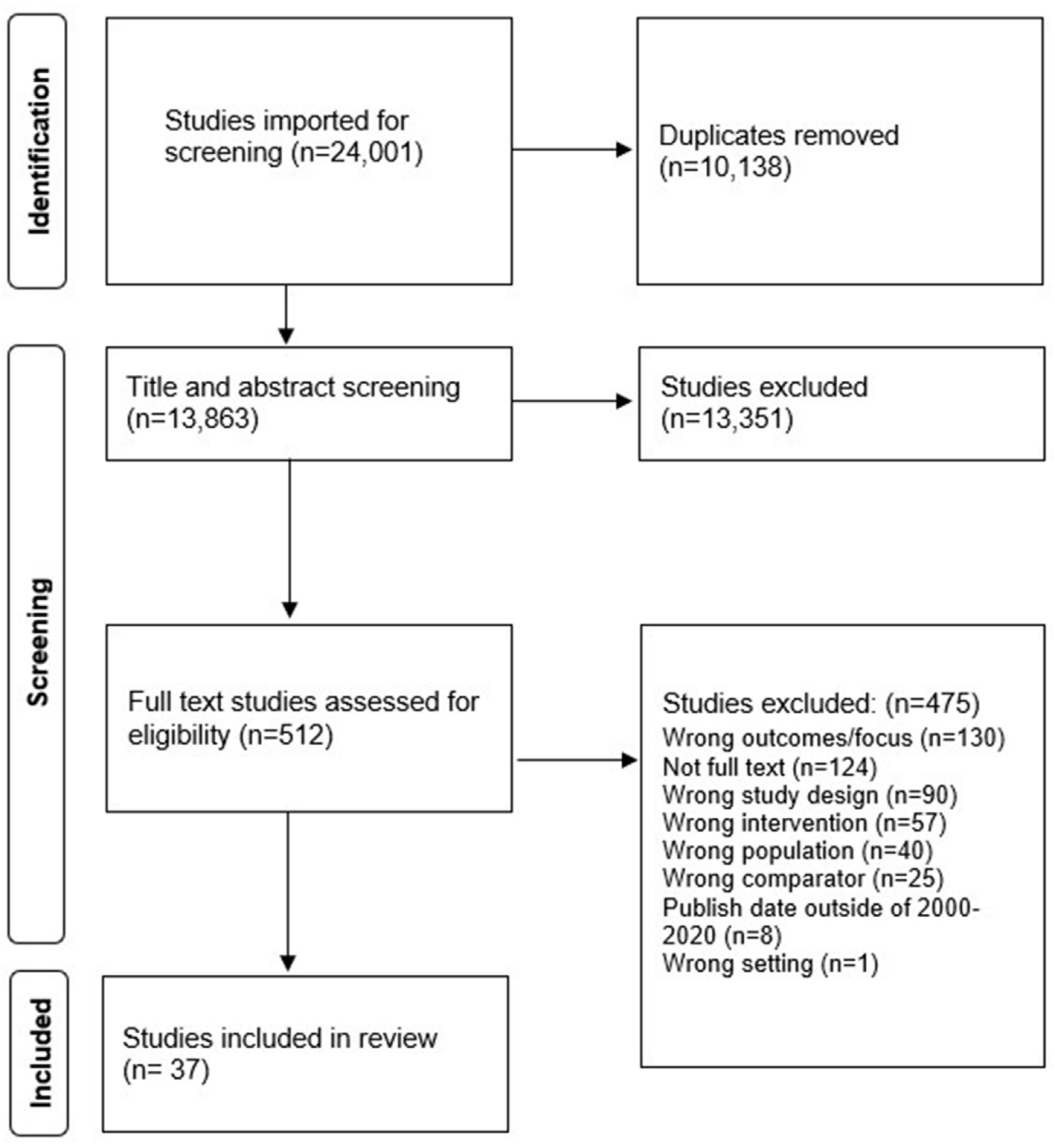
PRISMA flow diagram of the study identification and selection process for eligible articles. PRISMA, Preferred Reporting Items for Systematic Reviews and Meta-Analyses.

The search identified a total of 24,001 potentially relevant studies that were imported into Covidence for screening and review. After 10,138 duplicates were removed, the authors screened 13,863 titles and abstracts. We identified 512 potentially eligible studies for full-text review; a large number of articles progressed to full-text review as abstracts often did not include the detail necessary to discern whether an SVC model, with explicit local sourcing of food, was employed. Following full-text review, 37 articles representing 34 distinct studies were identified for inclusion. Common reasons for exclusions were wrong outcomes/focus (*n* = 130), lack of full text (i.e., abstract only) (*n* = 124), and wrong study design (*n* = 90). The PRISMA flow diagram (Figure 1) illustrates the selection process of articles for systematic review.

### Data type and study designs

Of the 37 articles that met our inclusion criteria, 12 reported on quantitative data [[43], [44], [45], [46], [47], [48], [49], [50], [51], [52], [53], [54]], 17 reported on qualitative data [[55], [56], [57], [58], [59], [60], [61], [62], [63], [64], [65], [66], [67], [68], [69], [70], [71]], and 8 employed mixed methods [[72], [73], [74], [75], [76], [77], [78], [79]]. However, of the 8 mixed-methods articles, only 2 met both the quantitative and qualitative inclusion criteria [73,76]. For the remaining 6 [72,74,75,[77], [78], [79]], only qualitative data were included; this was often because there was no control or comparison group for the quantitative analyses. Among quantitative analyses, the following study designs were used: RCTs (*n* = 3) [49,50,53], cluster-RCTs (*n* = 2) [45,52], quasi-experimental nonequivalent control group design [43,46], quasi-experimental time series with control group [54], quasi-experimental pretest-posttest with control group [44,51,73], quasi-experimental pilot [76], quasi-experimental evaluation (*n* = 1) [47], and randomized controlled feasibility study (*n* = 1) [48]. Qualitative data were collected via focus groups (*n* = 15) [[55], [56], [57],^59^,^60^,[62], [63], [64], [65], [66],^69^,^72^,^73^,^76^,^79^] and/or interviews (*n* = 13) [[57], [58], [59],^61^,^67^,^68^,^70^,^71^,[74], [75], [76], [77], [78]], with 6 described as “semistructured” [58,68,70,71,75,76], 3 as “in-depth” [57,59,61], 3 as telephone interviews [67,77,78], and 1 being an interviewer-led survey with open-ended questions [74].

### Methodologic quality of included studies

Of the 14 articles that included quantitative outcomes, methodologic quality was rated as “good” for 2 articles [47,52], “fair” for 7 articles [44,45,[48], [49], [50],^53^,^54^], and “poor” for 5 articles [43,46,51,73,76]. Among the 25 articles that reported qualitative data, methodologic quality was rated as “good” for 14 articles [55,56,58,59,[62], [63], [64], [65], [66],[68], [69], [70], [71],^75^], “fair” for 9 articles [57,60,61,67,[72], [73], [74],^76^,^79^], and “poor” for 2 articles [77,78]. The 2 mixed-methods articles that met both the quantitative and qualitative inclusion criteria [73,76] received 2 quality ratings, 1 for the quantitative methods and 1 for the qualitative methods, for a total of 39 quality ratings. Quantitative studies rated as “poor” were compromised by their lack of randomization, small sample size, high dropout rate (a “fatal flaw” per the tools used), or high loss to follow-up. Qualitative articles were given “poor” or “fair” ratings because of insufficient methodologic detail.

### Intervention types, components, and locations

The included articles were diverse in design and intervention type; thus, data are synthesized in Table 1 and narratively summarized by type of SVC model and studied outcomes. Of the included articles, SVC intervention types included the following: FM (*n* = 24) [43,46,47,50,51,[53], [54], [55],[58], [59], [60], [61], [62],[64], [65], [66],^68^,^70^,^71^,^74^,^75^,[77], [78], [79]], PRx (*n* = 7) [56,67,68,71,73,77,78], CSA (*n* = 5) [48,49,64,69,72], MM (*n* = 5) [45,52,56,63,65], FTS (*n* = 2) [44,76], FSt (*n* = 2) [57,61], and FH (*n* = 1) [47]. Some articles (*n* = 10) evaluated interventions that included >1 SVC model (e.g., PRx program where vouchers were redeemable at an FM) [47,56,59,61,64,65,68,71,77,78]. Fifteen articles were multimodal in that they leveraged an SVC model(s) in tandem with other complementary supports (e.g., nutrition education) [43,45,48,50,52,53,56,[67], [68], [69],[71], [72], [73],^76^,^78^]. Ten articles investigated nutrition incentive programs, such as Double Up Food Bucks (*n* = 3) [55,59,60] or WIC Farmers Market Nutrition Program (*n* = 4) [43,53,54,75]. These programs aim to increase the purchase of FVs by low-income consumers by providing incentives at the point of purchase [80]. The design of the included studies did not afford intervention dose comparisons.

For quantitative studies, the studied interventions ranged in duration from a single exposure [50,53] (e.g., a single education session) to 18 mo [47]. Of the 12 interventions that exceeded a single exposure, 3 (25%) were 2–3 mo in duration [43,54,73], 6 (50%) were 4–6 mo in duration [[44], [45], [46],^48^,^51^,^76^], and 3 (25%) were 11–18 mo in duration [47,49,52]. For qualitative studies, data were often collected at 1 time point to gain insight into established programs (e.g., FMs and CSAs). Studies were largely conducted in urban areas (*n* = 25) [43,44,[46], [47], [48],[50], [51], [52], [53], [54],[56], [57], [58],^60^,[62], [63], [64], [65], [66], [67], [68],^70^,^71^,[74], [75], [76],^78^] with only 4 studies focused solely on rural regions [49,61,73,77]. Three studies were conducted in both urban and rural areas [55,59,79], and the location was indiscernible for 2 studies [45,69,72].

### Nutrition education

Fourteen studies included a nutrition education component [43,45,48,50,52,53,56,[67], [68], [69],[71], [72], [73],^76^,^78^]. Seven studies employed in-person, group education [43,48,50,67,69,72,76]. Fours studies employed individual or passive education via 1 of 3 formats: 1-on-1 counseling [68,71], online lessons [53,73], and printed brochures[56]. Two studies employed a mix of individual and group education formats [45,52,78].

The intensity of nutrition education varied widely in frequency, length, and spread of sessions. The lowest frequency was 1 lesson at the start of the intervention[43,50,53], and the highest frequency was 22 lessons spread across the duration of the intervention [76]. Other reported frequencies included 3 [68,71], 4 [78], 6 [67], 9 [48,69], and 10 lessons [73]. The length of the education lessons varied by format. Online lessons were short (10–20 min) compared with in-person lessons, which were often reported to be 1 h in duration. Lessons were spread across the duration of the intervention and/or farmer’s market season and were often offered weekly, biweekly, or monthly.

Five studies leveraged existing curricula and dietary guideline resources from federal health agencies and professional associations, including the USDA Dietary and Physical Activity Guidelines for Americans [69,78], the national standards for Diabetes Self-Management Education [50], the Dietary Approaches to Stop Hypertension and Diabetes Prevention Program [73], and the Cook Smart, Eat Smart curriculum [48]. Four studies explicitly described the use of theoretical frameworks to guide the development of a study-specific curriculum [45,53,69,73], such as Social Cognitive Theory [53,69] and Adult Learning Theory [73].

For both online synchronous and in-person curricula, active learning components and cooking demonstrations were common. Field-based learning was employed in 2 studies and included tours of grocery stores, farms, and FMs guided by health educators [48,69]. Two studies described using a “tailored” nutrition education curriculum; Stotz et al., (2019) [73] tailored the curriculum to the cultural preferences, social needs, and educational needs of their target population, whereas the trial from which White et al., (2018) [69] drew their qualitative sample tailored the curriculum to the CSA season and availability of produce [81].

Two studies described offering their nutrition education sessions to families [69,78], and 1 study focused on children as the primary target [76]. Three of the 14 studies offered nutrition education lessons and materials in both the English and Spanish languages [50,52,53]. Reported educational materials include lesson handouts, produce information (e.g., purchasing, storing, and preparation tips), and recipe cards.

### Quantitative outcomes

Of the 14 articles that included quantitative outcomes, FV intake was the most commonly measured (*n* = 12) [[43], [44], [45], [46], [47], [48], [49], [50], [51], [52], [53], [54]], followed by anthropometric measures (e.g., BMI) (*n* = 4) [47,49,50,73], total diet quality (*n* = 4) [47,49,73,76], biomarkers of health (e.g., blood pressure) (*n* = 3) [49,50,73], and food security status (*n* = 2) [49,73]. Health outcomes (e.g., changes in chronic disease diagnoses) and QoL indicators were not measured in any of the included articles.

Of the 12 articles that measured FV intake, 7 found SVC intervention participation to significantly increase FV intake [[43], [44], [45], [46],^49^,^51^,^52^], 4 found no effect [48,50,53,54], and 1 found a negative effect (i.e., the comparison group had a significant increase in FV intake when compared with the intervention group) [47]. Among the studies that found a positive impact, improvements in FV intake were characterized differently depending on the methods and measures employed. Anderson et al., (2004) [43] used a structural equation model with a latent variable representing 3 measures of FV intake and found a regression coefficient of 0.33. Johnson et al., (2004) [51] used questions from the Behavioral Risk Factor Surveillance System and observed a 1.04-serving improvement in FV intake. Gans et al., (2018) [52] and Leone et al., (2018) [45] both used the National Cancer Institute’s FV screener and observed a 0.44 cup and 0.31 cup improvement, respectively. Herman et al., (2008) [46] and Berkowitz et al., (2019) [49] both employed 24-h recalls, but the former characterized their impact in terms of servings/1000 kcal consumed—observing an increase of 1.4 compared with controls—and the latter calculated Healthy Eating Index sub-scores, finding increased scores for total vegetables (+0.5), total fruit (+1.0), and whole fruit (+0.7) relative to controls. Finally, Kropp et al., (2018) [44] used plate waste data to study an FTS intervention and estimated a 0.06-serving improvement in vegetable intake.

Findings were generally null or mixed for the other outcomes. Four articles included anthropometric measures as an outcome, although none of them found an effect [47,49,50,73]. Two of the 4 articles that measured total diet quality found the SVC intervention to have no effect [47,73], whereas 2 found a significant increase in total diet quality among intervention participants [49,76]. Three articles assessed biomarkers of health (i.e., blood pressure [49,73], hemoglobin A1c [50,73], fasting blood glucose [73], and lipid panel [73]), but only 1 found an effect: a study in which the intervention group had a significant decrease in diastolic blood pressure [49]. Both articles that measured food security status found the interventions to have no effect [49,73].

### Qualitative findings

Among the 25 articles that reported qualitative findings, consumer barriers to SVC participation were reported in 24 articles [[55], [56], [57], [58], [59], [60], [61], [62],[64], [65], [66], [67], [68], [69], [70], [71], [72], [73], [74], [75], [76], [77], [78], [79]], and facilitators to participation were reported in 20 articles [55,[58], [59], [60], [61], [62], [63], [64],[68], [69], [70], [71], [72], [73], [74], [75], [76], [77], [78], [79]]. Although 12 articles examined FM engagement directly, 17 included FM as 1 part of the intervention (e.g., PRx vouchers to be redeemed at the local FM [55,[58], [59], [60], [61], [62],[64], [65], [66],^68^,^70^,^71^,^74^,^75^,[77], [78], [79]]). Other studies focused on PRx [67,68,71,73,77,78], MM [56,63,65], FSt [57], and/or FTS [76].

The most common barriers noted across all intervention types were insufficient program awareness, poor logistical access or convenience, and issues related to cultural incongruence. Participants reported lacking the knowledge necessary to fully utilize the SVC program outside the parameters of the study (for example, lack of clarity regarding outlet location, hours of operation, and available food assistance programming options). Further, participants reported poor logistical access or inconvenience, with specific concerns regarding the cost of produce, transportation limitations, and locations and/or hours that interfered with long working hours or busy family schedules. For interventions involving FMs and CSAs, participants reported dissatisfaction with the limited variety and reliability of produce available, especially relative to supermarkets. Spoilage of fresh produce was also a concern, often cited in tandem with tight food budgets. Many studies examined the utility of financial incentives, but logistical issues with voucher distribution and redemption were frequently reported.

Issues related to cultural incongruence were reported across 18 studies. This was expressed in several distinct ways. Most often, participants reported that fresh FV was either not routinely consumed or not a part of their traditional cultural foods. Among studies examining FM-based programs, experiences of bias (e.g., racial) were common and deterred regular participation. Experiences ranged from perceived bias against the presence of young children and language barriers to stigma associated with the use of food assistance programs and came from both vendors and other shoppers.

Common facilitators of SVC engagement included the health-promoting environment of SVC markets, feelings of community cohesion, financial incentives, and FV quality. The health-promoting environment was the most encompassing facilitator and involved opportunities for nutrition education (e.g., preparation and preservation techniques, recipes, and cooking skills) and social interaction, ultimately enhancing participants’ desire to eat more healthfully to prevent and manage chronic disease.

Community cohesion was identified as a facilitator distinct from the health-promoting environment, given that it was driven not by what the participants gained from the program but by how it enabled them to support their network and community. For example, participants appreciated the opportunity to interact and exchange information with FM vendors and CSA farmers and to support the local food economy. Others reported enjoying sharing nutrition education and excess FV with family and friends. Studies wherein health professionals facilitated the intervention, such as PRx models, reported that participants appreciated the collaboration between community resources.

Financial incentives were a commonly reported facilitator when available, although participants reported a resurgence of cost as a barrier as soon as the intervention concluded. Existing food assistance programs were more often discussed along with barriers, such as lack of awareness (e.g., how to use them at nontraditional markets), insufficient voucher amounts, and stigma related to program use.

Finally, the quality of FV available through SVC outlets was a key facilitator. Indeed, studies reported instances where participants were willing to put in extra time or effort to overcome barriers related to SVC engagement because of the high perceived quality of the available FV, especially relative to FV options available at local supermarkets or convenience stores.

Definitions of each barrier and facilitator, along with strategies to consider for enhancing future engagement, are outlined in Table 2. The applicability of each barrier and facilitator across studies is summarized in Table 3. Although strategies were not directly solicited from participants across all studies, several were reported based on their emergence during data collection.

**TABLE 2:** Emergent barrier and facilitator themes with reported strategies and considerations for supporting participant engagement in short value chain interventions

Category	Theme (study frequency)	Definition	Strategies and considerations
Barriers to SVC participation	**Lack of convenience (*n* = 22)**	Participants expressed **inability or unwillingness to use SVC outlets more often because of inconveniences** related to issues with transportation, location, time availability (i.e., work or family schedules conflicting with hours of operation), limited variety or reliability of FV or staple food options, and issues surrounding food assistance incentives. *Codes: transportation, location, operating hours, food assistance issues, variety, reliability*	Where feasible, **expand operating hours**, locate markets **near public transportation** or **consider delivery** options, reduce the burden of multi-stop food shopping, and optimize the implementation of incentives.
	**Lack of awareness (*n* = 21)**	Participants **lacked key information that was either necessary or helpful** in making the decision to use or not use SVC outlets more frequently outside of study participation. Examples include unawareness of locations or hours of operation, acceptance of food assistance programming vouchers, or perceived cost relative to other retail outlets. *Codes: Food assistance options, cost of produce, location, operating hours*	**Increase marketing and advertising** of locations and hours, food assistance programming available, and weekly product prices and availability.
	**Cultural incongruence (*n* = 18)**	Participants reported a range of perceptions and experiences, most commonly as **feelings of judgment or bias from either staff or other shoppers** in relation to language barriers, use of food assistance vouchers, or the presence of young children. This also included participant reports of cultural incongruence, i.e., **lack of familiar or preferred foods**, farmer hygienic presentation, and general unfamiliarity with the SVC method. *Codes: Unwelcoming environment, eating habits, and preferences*	Introduce more ethnic or culturally relevant foods; reduce the stigma associated with food assistance use; **expand and embrace cultural congruence as a social norm** through education.
Facilitators of SVC participation	**Health-promoting environment (*n* = 23)**	Participants referenced **health-promoting benefits of the shopping experience**, such as increased socialization, FV consumption or general healthy eating promotion (at individual and household levels), and **opportunities for nutrition education**. *Codes: Social environment, desire to eat healthfully, nutrition education opportunities*	**Increase opportunities for culturally tailored education** such as FV preparation and incorporation into meals, food safety, and general or disease-specific nutrition topics; **encourage family involvement** in interventions where possible.
	**Financial incentives (*n* = 17)**	Participants reported that financial incentives, whether existing **food assistance acceptance or experimental incentive intervention**, increased accessibility to FV. This also encompasses participants citing generally **lower produce prices** than other food retail outlets. *Codes: Food assistance programming, cost of produce*	**Provide convenient, user-friendly incentives** and **education to both shoppers and staff/vendors** on their utilization; **ensure incentive amounts meet the needs** of eligible participants.
	**Community cohesion (*n* = 16)**	Participants expressed a **broad desire to connect to the local community**, including engaging with farmers, family, and friends through the food procurement experience and supporting the local economy. Conversely, participants expressed gratitude and appreciation for the kindness and education received from vendors, staff, or other shoppers. Participant reports of support from the medical community to initiate these relationships are also included. *Codes: Connecting with farmers, friends, and family; medical community support*	**Promote SVC involvement as social and mutually beneficial** in nature; **leverage the medical community** to help bridge health gaps for patients by providing comprehensive care and connections with community resources.
	**FV quality (*n* = 13)**	Participants cited **higher quality FV in comparison to other food retail outlets** (e.g., grocery stores). Referenced qualities included superior taste, freshness, and terms such as "healthier," "organic," "natural," "local," and "home-grown." *Code: Produce quality*	**Raise awareness of comparative pricing** and quality between SVC and other retail outlets; **Use strategic marketing** to promote cost- and quality-value to potential customers.

Abbreviations: FV, Fruit and vegetable, SVC, short value chain.

**TABLE 3:** Occurrences of barrier and facilitator themes within included qualitative studies

			Barriers to SVC participation			Facilitators of SVC participation			
Author, y	Title	Intervention type	Lack of awareness	Lack of convenience	Cultural incongruence	Health-promoting environment	Financial incentives	Mutual aid in the community	Produce quality
Cahill et al., 2020 [67]	Qualitative research study on addressing barriers to healthy diet among low-income individuals at an urban, safety-net hospital	PRx	X	X		X			
Cohen et al., 2019 [60]	Facilitators and Barriers to Supplemental Nutrition Assistance Program Incentive Use: Findings From A Clinic Intervention for Low-Income Patients	FM	X	X		X	X	X	X
Colasanti et al., 2010 [79]	Understanding barriers to farmers’ market patronage in Michigan: perspectives from marginalized populations	FM	X	X	X	X		X	X
Cotter et al., 2017 [64]	Low-income adults’ perceptions of farmers’ markets and community-supported agriculture programs	FM, CSA	X	X	X	X			X
DeWit et al., 2020 [56]	Beyond clinical food prescriptions and mobile markets: parent views on the role of a healthcare institution in increasing healthy eating in food-insecure families	MM, PRx	X	X	X		X	X	
Di Noia et al., 2017 [66]	Perceived Influences on Farmers’ Market Use Among Urban, WIC-enrolled Women	FM	X	X	X	X	X		
Esquivel et al., 2020 [77]	Keiki Produce Prescription (kprx) Program Feasibility Study to Reduce Food Insecurity and Obesity Risk	PRx, FM				X	X	X	
Forbes et al., 2019 [78]	“Prevention Produce”: Integrating Medical Student Mentorship Into A Fruit And Vegetable Prescription Program for At-Risk Patients	PRx, FM	X		X	X	X	X	
Garner et al., 2020 [55]	A Qualitative Evaluation of Double Up Food Bucks Farmers’ Market Incentive Program Access	FM	X	X	X	X	X	X	X
Gibson et al., 2014 [76]	Farm-to-School, School to Home: An Evaluation of a Farm-to-School Program at an Urban Core Head Start Preschool Program	FTS	X	X	X	X		X	
Grace et al., 2007 [74]	Barriers to Using Urban Farmers’ Markets: An Investigation of Food Stamp Clients’ Perceptions	FM	X	X	X	X	X		X
Haynes-Maslow et al., 2015 [65]	Low-Income Individuals’ Perceptions About Fruit and Vegetable Access Programs: A Qualitative Study	MM, FM	X	X	X	X	X		
Headrick et al., 2021 [75]	Customers’ Views on the Implementation of a Farmers Market Incentive Program: Successes and Opportunities for Improvement	FM	X	X	X	X	X	X	
Horning et al., 2020 [63]	Full-Service Twin Cities Mobile Market Impact: Qualitative Findings From Focus Groups With Customers	MM	X	X		X	X		X
Hu et al., 2013 [57]	Community Perspectives on Barriers and Strategies for Promoting Locally Grown Produce From an Urban Agriculture Farm	FSt	X	X	X	X		X	X
Larimore, 2018 [58]	Cultural Boundaries to Access in Farmers Markets Accepting Supplemental Nutrition Assistance Program (SNAP)	FM	X	X	X	X	X	X	X
Masci et al., 2020 [59]	Double Up Food Bucks: A Qualitative Evaluation of Usage, Impact, Barriers, and Facilitators	FM, MM	X	X	X	X		X	X
McGuirt et al., 2014 [61]	Factors Influencing Local Food Procurement Among Women of Reproductive Age in Rural Eastern and Western North Carolina, USA	FM, FSt	X	X	X	X	X	X	X
McGuirt et al., 2019 [72]	A Mixed-methods Examination of the Geospatial and Sociodemographic Context of a Direct-to-Consumer Food System Innovation	CSA		X		X			
Savoie Roskos et al., 2017 [70]	Understanding the Experiences of Low-Income Individuals Receiving Farmers’ Market Incentives in the United States: A Qualitative Study	FM	X	X	X	X	X	X	X
Schlosser et al., 2019 [68]	“The coupons and stuff just made it possible”: economic constraints and patient experiences of a produce prescription program	PRx, FM	X	X		X	X		
Schlosser et al., 2019 [71]	“You Guys Really Care About Me…”: a Qualitative Exploration of a Produce Prescription Program in Safety-Net Clinics	PRx, FM	X		X	X		X	
Stotz et al., 2019 [73]	A Supplemental Produce and eLearning Nutrition Education Program for Georgians Who Use Safety-Net Clinics for Their Health Care	PRx		X	X	X	X		
Wetherill and Gray, 2015 [62]	Farmers’ Markets and the Local Food Environment: Identifying Perceived Accessibility Barriers for SNAP Consumers Receiving Temporary Assistance for Needy Families (TANF) in an Urban Oklahoma Community	FM	X	X	X		X	X	X
White et al., 2018 [69]	The perceived influence of cost-offset community-supported agriculture on food access among low-income families	CSA		X		X	X	X	X
Total occurrences across studies			**21**	**22**	**18**	**23**	**17**	**16**	**13**

Abbreviations: CSA, Community-supported agriculture; FM, farmers market; FSt, farm stand; FTS, farm-to-school; MM, mobile market; PRx, produce prescription program; SVC, short value chain; WIC, special supplemental nutrition program for women, infants, and children.

## Discussion

To our knowledge, this is the first systematic review to examine the impact of participant experiences with SVC models of healthy food access in the United States. The dual objectives, drawing on both quantitative and qualitative studies, afford a robust review from which we generate nuanced insights regarding the burgeoning scholarship on local food system models for advancing food and nutrition security and health equity. This review found mixed efficacy of SVC models, with improved FV intake being the most consistently demonstrated impact. SVC interventions vary widely in design, although FMs are more commonly studied than other intervention types. Despite such model variety, we found there to be a common set of barriers to and facilitators of participant engagement across model types.

### Quantitative findings

Among quantitative studies, FV intake was the most frequently measured outcome and 1 for which findings were generally promising. Other quantitative outcomes were sparsely measured or not measured at all. Even so, improving FV intake is a key, proximal mediator of longer-term health impacts; FVs encompass a wide array of foods that provide dietary fiber, vitamins, and minerals and are a source of phytochemicals that have numerous protective mechanisms [82]. Evidence indicates that higher intake of FV is associated with reduced risk of heart disease [83], stroke [84], lower mortality [85], and has a positive impact on mental health status in adults [86]. The impacts observed in the reviewed studies—namely those for which intake improved by ≥1 serving (or a half cup)—were clinically meaningful, which has far-reaching implications given that low-income, food-insecure households are at a heightened risk of chronic disease and mental health conditions[5,7,8,87].

Scholars and practitioners alike tend to be concerned about the long-term sustainment of impacts measured over relatively short-term studies. In the case of FV intake, follow-up studies have been done to assuage this concern. Marshall et al., (2020) [88] conducted a 2-y follow-up on a school-based intervention that increased child intake of FV and found a sustained and significant increase in participant intake compared with baseline. Neville et al., (2015) [89] also conducted an 18-mo follow-up of an RCT in older adults and observed long-term positive changes in FV intake. If we want to move beyond understanding the proximal impacts of such interventions, although, and discern for which models the proximal impacts translate into more distal impacts on chronic disease morbidity, studies of greater duration than was generally observed in this review will be necessary.

Anthropometric measures, total diet quality, health biomarkers, and food security status were assessed less frequently. This was a surprise, particularly the lack of food security data, as many studies cited this as a motivating concern and rationale for targeting low-income households. The dearth of such outcomes may be related to the burden incurred by both researchers and participants to collect it (with potential trade-offs for study retention) or the lack of changes to such outcomes in pretrial pilot studies (though this would be expected if pilots were relatively short in duration and underpowered, as is often the case). None of the included studies reported QoL or health outcomes. This may also be related to relatively short study durations or the participant burden associated with rigorous interventions, which may plausibly harm QoL [90]. This is something for researchers to consider, given the practical importance of QoL for participant well-being and the key role of health-related QoL data in cost-utility analyses comparing interventions toward evidence-based resource allocation.

### Qualitative findings

The aggregation of qualitative insights revealed insufficient program awareness, poor logistical access or convenience, and issues related to cultural incongruence as common barriers to participant engagement across intervention types. Ubiquitous facilitators of engagement included the health-promoting environment of SVC outlets, feelings of community cohesion, financial incentives, and FV quality.

Although identifying barriers, participants also offered strategies—often unsolicited—to enhance sustained participation in future programs. This reflects a broad interest in and commitment to improving SVC models among low-income individuals. Participants perceived seasonal FV nutrition education (i.e., preparation and storage methods) and community connection to be facilitators with unique applicability to SVC models and may be key points of focus for program administrators. Despite apparent interest in the studied models, this review revealed how a common set of barriers has persisted throughout the 20-y review period. Given the ubiquitous and persistent nature of these barriers, further research on such barriers and facilitators may be less impactful than efforts to understand and test implementation solutions. Of note, there was substantial interdependence between barriers and facilitators; the decision by low-income households to use an SVC model is multifaceted, weighing economic, logistical, and sociocultural factors beyond individual control. This suggests that systems-level interventions may be more effective than singular or isolated approaches. Addressing barriers in the long term requires sustained cross-sector partnerships and multimodal interventions that address the interplay between program access, awareness, cultural congruence, and financial incentives.

### Multimodal interventions

Financial and self-efficacy barriers create distinct and well-documented challenges to achieving a nutritious diet [[91], [92], [93]]. This review included 15 multimodal interventions that aimed to mitigate these 2 barriers simultaneously. Modalities for enhancing financial access to FV included the provision of FM coupons or free or discounted produce. Modalities to support diet-related self-efficacy included nutrition lessons, cooking skill workshops and demonstrations, and educator-guided tours of FM and grocery stores.

Evidence suggests that multimodal interventions have a greater likelihood of affecting health behavior change compared with unimodal interventions [94,95]. Multimodal interventions are particularly recommended for interventions targeting household-level changes (as is common for SVC interventions) and for managing common and complex health conditions, such as obesity, diabetes, and cancer [[94], [95], [96], [97]]. In this review, 8 of the 15 multimodal interventions reported on our quantitative outcomes of interest [43,45,48,50,52,53,66,76]. Of those, only 4 showed significant positive changes—for the outcomes of diet quality [76] and FV intake [43,45,52]. Examination of the modalities used in these interventions revealed 2 characteristics that seem key to facilitating successful outcomes: social marketing and intensive nutrition education.

Social marketing strategies appear to be a poignant mechanism for increasing awareness of SVC programs, encouraging engagement, and promoting a sense of community. Sharpe et al., (2020) [47] concluded that improving spatial access to healthy foods alone was ineffective in improving diet quality among disadvantaged communities living in USDA-defined Low-Income Low-Access areas and suggested a multifaceted approach focused on barriers experienced by the target community. Incorporating promotional activities in intervention design can be particularly advantageous, as a lack of awareness about the existence and operations of SVC programs (including location, hours, and acceptance of Electronic Benefit Transaction) was reported as a barrier in 11 of the 25 qualitative studies in this review [55,[57], [58], [59],^61^,^62^,^66^,^70^,^74^,^75^,^79^]. Gibson et al., (2014) [76] engaged parents in a 6-mo FTS intervention via monthly in-school FM displays with free seasonal produce and printed recipes. Leone et al., (2018) [45] and Gans et al., (2018) [52] leveraged reduced-price mobile fresh market models and consistently marketed throughout the duration of the interventions (6 and 12 mo, respectively). Their strategies included visually attractive newsletters delivered regularly via mail and email (weekly and monthly) with market information and invitations to join intervention-related community events (e.g., cooking demonstrations, taste-testing, and prize raffles, respectively).

A second mechanism for driving positive outcomes appeared to be the implementation of a dynamic nutrition education curriculum that offered frequent lessons, promoted both knowledge and skills, incorporated field-based learning activities, and, most importantly, was tailored to produce seasonality. Although a general lack of nutrition knowledge and cooking skills has been reported as a barrier to increasing FV intake[57,65,73], unfamiliarity with FM and CSA produce items were specifically mentioned as a barrier to engagement in numerous studies [10,62,64,69,77]. In Gibson et al., (2014) [76], weekly nutrition lessons included farmers serving as guest speakers, cooking classes, a field trip to the grocery store, and school-based gardening. In Leone et al., (2018) [45] and Gans et al., (2018) [52], all intervention elements were focused on in-season produce and included content on key nutrients, health benefits, relevant recipes, and tips for selection, storage, and time-efficient and budget-friendly approaches for preparation and integration in the diet.

### Unimodal interventions

Unimodal interventions were far less common than multimodal interventions in this review. Those that were included, although, were generally effective; 4 of the 5 unimodal interventions reported significant positive changes in FV intake [44,46,49,51], diet quality [49], and diastolic blood pressure [49]. Two of the 4 unimodal interventions provided the highest participation incentives of all reviewed studies, which may have helped to drive engagement (and impact) by compensating effectively for the time and resource scarcity experienced by many participants [57,61,[65], [66], [67], [68],[71], [72], [73],^78^,^79^]. Herman et al., (2008) [46] offered $240 in produce vouchers, dispensed biweekly in $20 increments over 6 mo, and Berkowitz et al., (2019) [49] offered $600 toward CSA shares divided over 2 growing seasons. In the remaining 2 interventions, Johnson et al., (2004) [51] resolved the persistent transportation barrier [55,56,58,60,64,[66], [67], [68],^71^,^73^,^75^] by delivering FM baskets to homebound seniors on a biweekly basis for 5 mo, and Kropp et al., (2018) [44] bypassed various access and affordability barriers via a FTS program in schools with high percentages of children from low-income families.

### Financial incentives

Financial incentives have been found to influence short-term dietary behavior change positively. When used as a catalyst for change rather than a reward, as is the case for the SVC models in this review, financial incentives can aid in the long-term maintenance of dietary behaviors [98]. Seventeen interventions (19 studies) described using financial incentives as part of the studied SVC model [43,46,49,50,[53], [54], [55], [56],^59^,^60^,[66], [67], [68],[70], [71], [72],^75^,^77^,^78^]. Of those, 8 were FM-based and affiliated with a federally-funded nutrition assistance program: 4 WIC Farmers Market Nutrition Program [43,46,53,54,66] and 4 SNAP-Double Up Food Bucks [55,59,60,70]. PRx, CSA, and MM interventions also offered financial incentives. Incentives amounts ranged from $5 one-time vouchers [56] to $600 toward a multiyear CSA share [49]. The incentive value sufficient to motivate behavior change remains a subject of debate, especially in underserved communities where the trade-off of precious time and scarce resources can deem small incentives futile. A systematic review of financial incentives for dietary behavior change estimates $40 as an optimal starting incentive for weight management programs [98]. Six of the 19 studies reported quantitative impacts [43,46,49,50,53,54], with 3 demonstrating a significant increase in FV intake [43,46,49] and 2 of these being the aforementioned studies that offered the highest incentive amounts of all reviewed studies [46,49]. The federally-funded GusNIP supports the implementation of high-reach, low-intensity community projects and low-reach, high-intensity projects for underserved communities, with intensity being inclusive of services, nutrition education, and incentive amounts. Budgets allocated toward direct incentives by GusNIP grantees increased from 68.5% to 74.7% in a span of 1 y [99,100], suggesting a greater realization of the role of incentives in achieving program objectives. Future research on incentive amounts, with consideration of community characteristics and environmental context, may yield useful guidance on optimal incentives across SVC intervention types.

### Demographics and geographic location

Our systematic review findings highlight several shortcomings worth discussing. Each included article reported a higher percentage of female participants than males. In fact, 6 articles had only female participants, and 9 had >80% female participants. This can be partially explained by the number of studies that focused solely on WIC-related programs. However, having a higher proportion of female participants is not uncommon in nutrition, health, and food security-related research; as scholars work to understand mechanisms for enhancing population-wide health equity, this will be something to consider more purposefully during study planning and recruitment.

Additionally, most studies were focused on adults. Future studies need to consider the complex household dynamics and whether it would be prudent to enroll more than a single individual. Family-based multimodal interventions are favorable for the management and/or treatment of chronic health conditions in both adult and children populations compared with standard-of-care interventions [94].

Geographic representation is another area for which researchers will need to be more intentional moving forward. Studies in this review were largely conducted in urban areas. Pillar 1 of the White House National Strategy on Hunger, Nutrition, and Health calls for special attention to rural health, given the persistent structural inequalities (e.g., transportation issues) and disparities in food access that they face [18]. In 2021, 9 out of 10 counties with the highest food insecurity rates were rural [101]. This represents a major public health problem, given that 46 million Americans live in rural areas [102]. The USDA and NIH have also heightened their focus on rural health, signaling the importance and necessity of this in future research [103,104].

### Methodologic considerations

Assessing the risk of bias for the 37 articles that met our inclusion criteria presented some challenges. The tool used to appraise qualitative studies, the SRQR, offers well-defined standards for reporting qualitative data; however, it was challenging to use this tool for pragmatic qualitative studies. For example, qualitative research undertaken with a practical or clinical orientation scored less favorably for criteria emphasizing explicit discussion of the research paradigm (e.g., postpositivist or constructivist) and elements related to researcher positionality (e.g., researcher characteristics and practices demonstrating reflexivity). Relying on the SRQR as a tool for assessing rigor thus required the research team to interrogate all other elements of the pragmatic studies more thoroughly to determine a reasonable risk of bias assessment rating. This suggests that there are opportunities to enhance the rigor and transparency with which qualitative inquiry is pursued, even when approached pragmatically or as 1 part of a mixed-method effort. An additional challenge arose when assessing the rigor of qualitative studies because of a wide variation in how facilitators and barriers to SVC participation were reported and framed (e.g., the degree to which themes were explicit).

The National Institute of Heart, Lung, and Blood Quality Assessment Tools worked well for evaluating all quantitative studies. A notable exception arose when trying to determine how to use the tools for quasi-experimental designs that did not fit the parameters of controlled intervention studies, observational cohort and cross-sectional studies, or case-control studies. Ultimately, the study in question was excluded from the final review because it did not meet inclusion criteria. As quasi-experimental designs and econometric analyses—such as the differences-in-differences design leveraged by Olsho et al., (2015) [105]—gain traction in the health policy literature given their ability to estimate causal impact in the absence of RCTs (e.g., when such designs are unethical or impractical), multidisciplinary teams conducting systematic reviews need to consider the value of employing cross-disciplinary tools appropriate for more diverse study designs.

Very few of the studies that met inclusion criteria, in fact, were RCTs (*n* = 3) or cluster-RCTs (*n* = 2). It is unclear whether this was merely reflective of our inclusion criteria or if there are issues of feasibility when seeking to conduct an RCT to assess the effectiveness of SVC models. This may also connect to broader conversations about the questions and interventions for which RCTs are indeed appropriate and ethical to use, particularly if the target population includes structurally marginalized groups that may benefit from a minimal level of access to a particular SVC intervention.

### Articles published after 2020

Although 8 articles representing 7 distinct studies [[106], [107], [108], [109], [110], [111], [112], [113]] were published after 2020 and therefore excluded from the review based on our PROSPERO-registered protocol, the authors deemed it crucial to review these studies given the rapidly expanding literature on local food system interventions. In contrast to the division of quantitative and qualitative analyses (14 compared with 25) represented in our review, 7 of the 8 articles published after 2020 collected only quantitative data [[106], [107], [108],[110], [111], [112], [113]]. Three studies were FM interventions [107,108,110,111]; 2 were PRx interventions [106,112]; 1 was an MM [109]; and 1 was a FH intervention [113]. Six of the 8 articles included FV intake as an outcome [107,108,[110], [111], [112], [113]]; 3 included anthropometric outcomes (BMI) [[106], [107], [108]]; and 1 included biomarkers of health (hemoglobin A1c) [106]. This suggests that scholars continue to leverage FV intake as a measure of impact while also enhancing the state of science via the study of anthropometrics and health biomarkers.

The 1 qualitative study examined a MM intervention [109] and identified similar barriers to and facilitators of engagement as those studies included in the review (i.e., poor logistical access but helpful financial incentives). These barriers and facilitators were collected via a concept mapping activity with intervention participants; such an activity would not have met our methodologic inclusion criteria but is in line with emerging best practices for community-engaged data collection. Future qualitative reviews will want to keep this in mind.

The design of the quantitative studies—3 RCTs [106,107,110], 1 process evaluation of an RCT [108], and 3 quasi-experimental designs[[106], [107], [108]]—included randomization more often than did the studies included in our review. This is an encouraging trend toward high-quality quantitative study designs, suggesting that this area continues to be a compelling avenue of research and that concerns regarding the applicability of randomly assigned study designs to SVC models may be for naught. The relative dearth of qualitative studies since 2020 is concerning; continuing to integrate the qualitative perspectives of study participants is core to optimizing the implementation of these models, especially as they get scaled to new settings and culturally distinct populations.

### Policy and research considerations

Pandemic-era media coverage and the recent White House Conference on Hunger, Nutrition, and Health generated robust public and private commitments to addressing food and nutrition insecurity in the United States. With the 2025 United States Dietary Guidelines committee at work and discussions commencing regarding the next Farm Bill, the country is at a critical juncture regarding how we will address the complex interface of food supply chains, nutrition, and health. This review highlights critical opportunities to bolster our understanding of how SVC models may be leveraged to advance national goals at the interface of agricultural, economic, social, biological (nutrition), and health care systems.

Specifically, we contend that any federal investments in this domain should include sufficient resource allocation for robust, nationally coordinated evaluation (as has been facilitated for GusNIP grantees). The central goal would be to interrogate the potential of SVC models using consistent methods and measures for programs implemented across the rural-urban continuum and among diverse communities via longer-term studies focused on measurable health impacts. Studies focused on understanding and testing implementation strategies designed to overcome known (persistent) barriers and maximize impacts—for participants and across the value chain—will also be key.

For scholars and practitioners working together to design, implement, and study SVC models, we encourage consideration of how social marketing and engaging, self-efficacy-enhancing forms of nutrition education could be employed and tested, given our findings regarding their role in successful interventions to date. We also encourage the explicit study of intervention dose, including the value of any financial incentives; engagement and study of household units, not just individuals; and the inclusion of rural and remote contexts. Together, the insights from these more strategically designed studies can balance the need for evidence-based public health investments with ever-present concerns regarding resource limitations.

## Acknowledgments

We thank research librarian Anna Biszaha, Masters of Library and Information Science (MLIS) of the Health Sciences Library at Ohio State University, for her thorough and kind assistance with formulating the search string and its adaptation for each database.

### Author contributions

The authors’ responsibilities were as follows – JAG, HH: conceptualized the review and protocol; AB: conducted the search in each database; HH, AB, KIP, KG, JAG, KKG: screened titles and abstracts for eligibility and inclusion using Covidence; KG, KKG, KJ, KIP, ECK, EL, YL, KA-M, HH: extracted data from all included studies; KG, KKG, KA-M, KIP, KJ, ECK, EL, YL: evaluated each study’s risk of bias; ECK: coded the results section of all qualitative studies to support thematic development; KG, KKG, KA-M, ECK, KJ, EL, YL, JAG: engaged in discussions and decisions regarding data synthesis; HH, KG, KKG, KA-M, ECK, JAG: contributed original writing to the draft; all authors reviewed the draft for intellectual content, read, and approved the final manuscript.

### Conflict of interest

The authors report no conflicts of interest.

### Funding

The authors reported no funding received for this study.

### Data Availability

No data were generated via this review.
