# Supplementary material for: Local Food System Approaches to Address Food and Nutrition Security among Low-Income Populations: A Systematic Review
Source: Adv Nutr. 2024 Mar 11;15(4):100156. doi: 10.1016/j.advnut.2023.100156 (PMC11031423; doi:10.1016/j.advnut.2023.100156)
Supplement: Multimedia component 1 [file mmc1.docx]

Local Food System Approaches to Address Food and Nutrition Security among Low-Income Populations: A Systematic Review

Katharine Garrity et al.

Appendix A: Search Strategy

Search terms and strings were created under the expert guidance of a research librarian. A drafted search string was provided to the librarian who then refined and modified the string to function with a variety of online databases. The topics of this review were distilled into three domains: (1) disparity, (2) short value chains (SVCs), (3) food. The analysis of these domains and the terms chosen to describe them as well as other considerations regarding the search terms are documented below.

*Disparity*

Keywords describing socioeconomic factors and low-income populations were used in this domain. The general term “economics” was not included due to the copious number of articles it returned in the preliminary search and the lack of relevant articles found in relation to this review. Words to describe food assistance programs aimed at this population, such as “food stamps”, “Women Infants & Children”, “Supplemental Nutrition Assistance Program” were also included. These terms were added after finding a small number of articles in the preliminary search phase that did not reference general terms to describe low-income populations but focused on food assistance programming. While the terms “food insecurity” and “food insecure” do describe an outcome of interest in this review, their existence is enmeshed with socioeconomic inequality, and therefore were included as search terms. The term “food desert” was not included in this search because the aim of this review was to assess the impact of utilizing SVC models of healthy food access, not to explore the concept of a lack of access to healthy foods at the community level.

*Short Value Chains*

SVC models proved to be the most complex domain to finalize within this search. This domain initially included the term “farm(s),” due to the prominent role farms have in SVC models. Following the addition of terms relating to farm to school programs and their variations (ie. farm to preschool, farm to table, farm to plate, etc.), it was decided the general term “farm(s)” would be removed. “Farm to school” and its variations were not initially included in the drafted search string; however, these concepts do have the capability to function as SVC models and therefore were included. Examples of SVC models such as “buying club(s),” “food hub(s),” “community support agriculture,” “farmers market(s),” etc., were all terms added to this domain. Additionally, terms describing incentive programs such as “financial incentive,” “nutrition incentive,” and “incentive program,” as well as their method of alternative currency (i.e., “token”, “voucher”, “subsidy”) were included. While incentive programs do not exclusively exist as SVC models, these programs aim to reach low-income populations and can have a reduced number of intermediaries (e.g., as is the case with farmers market incentive programs and cost-offset community supported agriculture). Therefore, it was important to include these terms. Despite the lack of a clear definition of “local food,” terms related to such were added to this domain given the importance of reducing distance between producer and consumer in the concept of SVCs.

*Food*

The preliminary search string was created without food-related terms but was added after careful consideration. The addition of this concept allowed the authors to expand the search to include a variety of incentives and programs while remaining focused on relevant food-centric programs. Terms related to “food”, “diet”, “nutrition”, “fruits”, and “vegetables” were included in this domain.

*Other*

Although this review was focused on articles regarding SVC models implemented in the United States only, geographical terms were not added to the search. There was concern that the addition of these identifiers might further complicate the terms list, given that each individual state might need to be added as a term, not just the country. In addition, there was concern that geography might not be specified until the full-text review stage and that such a complex search may unnecessarily limit the articles included in the review.

Databases in which to run the literature search were carefully considered. Agriculturally focused databases such as Agricola and CAB Abstracts were included given the emphasis in this review on SVC models of healthy food access. Public Affairs Index was included as well due to the impact the national policy environment can have on SVC model viability. The remaining databases were applicable to the medical and public health outcomes of focus.

PubMed Search String Used

(socioeconomic factors[mesh] OR socioeconomic[tw] OR socioeconomics[tw] OR poverty[tw] OR low income[tw] OR low resource[tw] OR poor[tw] or disadvantaged[tw] OR needy[tw] OR underclass[tw] OR vulnerable[tw] OR underserved[tw] OR under-served[tw] OR food insecure[tw] OR food insecurity[tw] OR food assistance[mesh] OR food assistance[tw] OR nutrition assistance[tw] OR food stamp[tw] OR food stamps[tw] OR supplemental nutrition assistance program[tw] OR snap[tw] OR (temporary assistance[tw] AND needy families[tw]) OR tanf[tw] OR women infants and children[tw] OR wic[tw])

AND

(short value chain*[tw] OR short supply chain*[tw] OR short food supply chain*[tw] OR sfsc[tw] OR local food system*[tw] OR local produce[tw] OR community supported agriculture[tw] OR csa[tw] OR csas[tw] OR co-csa[tw] OR co-csas[tw] OR farm share*[tw] OR farm to consumer[tw] OR farm to school[tw] OR farm to preschool[tw] OR farm to early care[tw] OR farm to ece[tw] OR farm to table[tw] OR farm to fork[tw] OR farm to plate[tw] OR farmers market*[tw] OR farmer’s market*[tw] OR mobile market*[tw] OR produce market*[tw] OR vegetable market*[tw] OR fruit market*[tw] OR produce stand[tw] OR produce stands[tw] OR vegetable stand[tw] OR vegetable stands[tw] OR fruit stand[tw] OR fruit stands[tw] OR farm stand[tw] OR farm stands[tw] OR produce prescription*[tw] OR vegetable prescription*[tw] OR food prescription*[tw] OR script[tw] OR scripts[tw] OR rx[tw] OR subsid*[tw] OR incentiv*[tw] OR voucher*[tw] OR coupon*[tw] OR token*[tw] OR buying club*[tw] OR food hub*[tw] OR produce program*[tw] OR vegetable program*[tw] OR fruit program*[tw] OR fv program*[tw] OR f v program[tw])

AND

(food supply[mesh] OR food[mesh] OR food[tw] OR fruit[tw] OR fruits[tw] OR vegetable[tw] OR vegetables[tw] OR veggie[tw] OR veggies[tw] OR diet[mesh] OR diet[tw] OR diets[tw] OR dietary[tw] OR nutrition[tw] OR nutritional[tw])

Appendix B: PICO Screening Guide

| **Participants** | - Within the United States - Households considered low-income   - as defined by <185% of the current Federal Poverty Level or   - as indicated by the authors of the reviewed articles - Exclusion: None |
| --- | --- |
| **Intervention** | - Short value chain models designed to improve healthy food access^1^ |
| **Comparisons** | - Households with no vs some exposure to an applicable intervention^2^ - For qualitative studies, there will be no applicable comparator. |
| **Outcomes** | **Primary**   - food security status^3^ - fruit and vegetable intake^4^ - total dietary quality^5^   **Secondary**   - Anthropometric measures^6^ - Biomarkers of health^7^ - Health outcomes^8^ - Quality of Life indicators^9^   **Qualitative**   - Consumer barriers to SVC participation - Consumer facilitators to SVC participation |
| **Study Design** | Quantitative – Any interventional study design, including:   - RCTs - Non-randomized trials - Pre-post studies - Quasi-experimental studies   Qualitative   - Focus group - In-depth interviews |

1. For the purpose of this work, a “short value chain model” will include any approach that:
   1. minimizes disconnect between farms and consumers by reducing one or more ‘middle’ portions of the traditional food supply chain, and
   2. leverages local or regional sources of healthy food (e.g. fruits and vegetables).
   3. Such models may include, but will not be limited to, farmers markets, local buying clubs, community supported agriculture, and food hubs.
      1. Gardening interventions will not be included
2. When data allows, we will also assess the role of intervention dose (e.g. no versus low versus high exposure to the intervention).
3. As measured by any version of the USDA ERS food security survey, The Hunger Vital Sign^TM^, one-item hunger screening question (Kleinman et al.), or one-item question in SEEK screener (Lane et al.)
4. As measured by the NCI Fruit and Vegetable Screener or other validated measure.
5. As measured by the Healthy Eating Index.
6. Such as BMI, weight, waist circumference.
7. Such as blood pressure, total cholesterol, HDL cholesterol, cholesterol ratio, triglycerides, fasting glucose, hemoglobin A1C.
8. Such as chronic disease diagnoses.
9. As measured by WHOQOL-BREF, QOL10, or other validated measure.
